# Outcome comparison of arthroscopic latissimus dorsi tendon transfer and muscle advancement for irreparable rotator cuff tear: a systematic review

**DOI:** 10.1097/JS9.0000000000002848

**Published:** 2025-07-08

**Authors:** Jun Lang, Vivek Kumar Morya, Yong-Beom Lee, Kyu-Cheol Noh

**Affiliations:** a School of Medicine, Hallym University, Chuncheon, Republic of Korea; b Department of Orthopedic Surgery, Hallym University Dongtan Sacred Heart Hospital, Hwaseong-si, Republic of Korea; c Department of Orthopedic Surgery, Hallym University Sacred Heart Hospital, Anyang-si, Republic of Korea

**Keywords:** arthroscopy, latissimus dorsi tendon transfer, muscle advancement, rotator cuff tear, shoulder surgery

## Abstract

**Purpose:**

To compare the clinical outcomes and complications between latissimus dorsi tendon transfer (LDTT) and muscle advancement (MA) for irreparable rotator cuff tears (IRCTs).

**Methods:**

A PRISMA-guided systematic review included 24 studies (956 shoulders: 750 LDTT, 206 MA) from MEDLINE, Embase, and Cochrane Library (searched October 2024). Eligible studies involved arthroscopic-assisted procedures for Patte stage 3 tears with Goutallier stage 3–4 fatty degeneration and ≥12-month follow-up. The analyzed outcomes included functional scores (Constant-Murley, UCLA, ASES), pain (VAS), acromiohumeral distance (AHD), range of motion (ROM), and complications. Risk of bias was assessed using the ROBINS-I and Cochrane tools, and statistical synthesis employed RevMan and R.

**Results:**

In this analysis, 24 studies (1 RCT, 13 cohort, 10 case series) involving 956 shoulders were included: 206 in the MA group (mean age 64.6 years, mean follow-up 19.7 months) and 750 in the LDTT group (mean age 60.8 years, mean follow-up 31.2 months). Both techniques resulted in significant functional improvement. Comparative analysis revealed no significant differences in the pooled mean improvements for the Constant-Murley Score, UCLA score, ASES score, VAS pain, forward flexion, or abduction, however, the LDTT group demonstrated significantly greater improvement in external rotation. The MA group experienced significantly higher rates of total complications (25.7% vs. 18.0%, *P* = 0.0206) and failure/retear/reoperation (20.8% vs. 8.9%, *P* = 0.0003). The rates of infection, nerve palsy, and stiffness were comparable between groups. Significant heterogeneity was observed in most continuous outcomes.

**Conclusions:**

LDTT and MA effectively restore shoulder function in IRCTs; however, their mechanisms differ. The LDTT excels in dynamic biomechanical compensation for external rotation, whereas MA achieves superior static joint stability. LDTT’s lower retear rates and higher complication risks associated with MA highlight the need for patient-specific surgical selection.

## Introduction

Rotator cuff tears are among the most frequent causes of shoulder pain and disability, with affected individuals often experiencing limitations in daily activities, severe sleep disturbances, and significant functional impairments^[1-3]^. As the incidence of these injuries continues to rise, they impose a growing burden on healthcare systems in terms of both direct treatment costs and lost productivity^[4-6]^. Surgical repair is the standard treatment for rotator cuff tears; however, a substantial subset of cases, particularly those classified as irreparable rotator cuff tears (IRCTs), cannot be treated using conventional repair techniques. These irreparable tears, often associated with tendon retraction, muscle atrophy, or advanced degeneration, affect nearly one-third of the patients undergoing surgery^[7-9]^.

**HIGHLIGHTS**

- First systematic review comparing latissimus dorsi tendon transfer (LDTT) and muscle advancement (MA) for irreparable rotator cuff tears.
- LDTT shows superior external rotation gains and lower retear rates, while both techniques provide comparable pain reduction.
- MA achieves superior static joint stability but carries higher overall complications and retear risks.

Management of IRCTs is particularly challenging in nonarthritic younger patients or active older adults for whom reverse total shoulder arthroplasty may be contraindicated or undesirable^[10–12]^. Consequently, surgical techniques aimed at preserving joint integrity, such as partial repair, subacromial spacers, superior capsular reconstruction (SCR), and tendon transfers, have gained prominence in recent years^[9,11–14]^.

Latissimus dorsi tendon transfer (LDTT) has emerged as a pivotal option. Initially described by Gerber *et al*, the LDTT was later refined to incorporate arthroscopic techniques, minimize deltoid muscle damage, and enhance outcomes^[15–17]^. The procedure involves transferring the latissimus dorsi tendon from its original insertion on the humeral shaft to the greater tuberosity of the humerus. This transfer alters the biomechanical role of the muscle from an internal to an external rotator, thereby restoring posterior force couples of the glenohumeral joint^[18]^.

Similarly, muscle advancement (MA), first introduced by Debeyre *et al*, focuses on mobilizing the supraspinatus and infraspinatus to enhance tendon excursion and improve repair potential^[19]^. Arthroscopically assisted and fully arthroscopic MA techniques have been developed in recent years, offering reduced surgical trauma and potentially lower complication rates^[20–23]^. Despite these advancements, limited comparative data exists regarding the outcomes of MA and LDTT, particularly in terms of functional recovery, radiographic metrics, and complications.

This systematic review aimed to address this gap by comprehensively analyzing and comparing the clinical outcomes of these two techniques for managing IRCTs. To ensure transparency in the reproducibility in the use of artificial intelligence in this study, we adhered to the recommendations outlined in the Transparency in the Reporting of Artificial Intelligence (TITAN) guidelines^[24]^.

## Materials and methods

**Figure 1.**
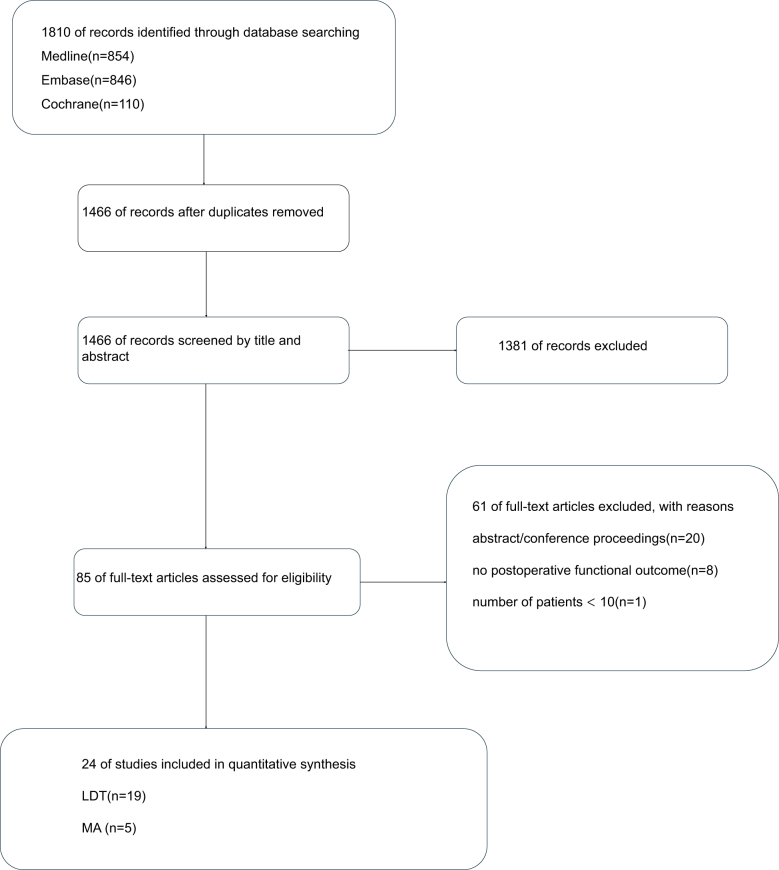
PRISMA (Preferred Reporting Items for Systematic Reviews and Meta-Analyses) study selection flow diagram.

This systematic review and meta-analysis of surgical treatments for IRCTs followed PRISMA guidelines (Fig. 1). Two authors independently screened the titles and abstracts, and a third senior author resolved the major discrepancies. The study protocol was registered with PROSPERO.

## Search strategy

A literature search was conducted across the MEDLINE, Embase, and Cochrane Library databases in October, 2024. The search terms included:

((latissimus dorsi) OR (latissimus dorsi tendon transfer)) AND (rotator cuff)

((muscle advancement) OR (muscular advancement)) AND (rotator cuff)

Specific search strategies were developed for each database (Supplemental Digital Content, available at: http://links.lww.com/JS9/E631).

## Eligibility criteria

The inclusion criteria were as follows: (1) Patte stage 3 posterior superior cuff tears with Goutallier stage 3 or 4 fatty degeneration; (2) patients undergoing arthroscopically assisted LDTT or MA procedures; (3) minimum 12-month follow-up; (4) sample size ≥10; (5) peer-reviewed publications with full text in English; and (6) outcomes assessed using the Constant-Murley Score (CMS), University of California-Los Angeles (UCLA) shoulder score, American Shoulder and Elbow Surgeons (ASES), acromiohumeral distance (AHD), visual analogue scale (VAS), or range of motion (ROM). Exclusion criteria were as follows: (1) insufficient procedural details; (2) duplicate patient populations; (3) nonclinical studies; (4) cases with glenohumeral osteoarthritis or Hamada grade ≥4 arthropathy; and (5) case reports, reviews, pilot studies, unpublished works, editorials, or conference materials. These criteria ensured the availability of high-quality relevant data.

## Data extraction and quality assessment

Two researchers reviewed the studies and extracted data on authorship, publication year, study quality, surgery type, demographics, and outcomes, including the UCLA shoulder score, CMS, ASES, AHD, and ROM (forward flexion FF, abduction, and external rotation ER). Quality was rated using the ROBINS-I for cohort studies and Cochrane’s risk of bias tool for randomized controlled trials (RCTs)^[24,25]^.

## Statistical analysis

Distinct meta-analytic methods were used to assess the MA and LDTT. For continuous clinical outcomes, pre-post mean differences within groups were synthesized from individual studies, and these effects were consistently pooled using a random-effects model^[26]^. To evaluate the robustness of these pooled effects for continuous outcomes, sensitivity analyses were conducted using RevMan 5.4, specifically through leave-one-out analysis. These analyses systematically varied the key assumptions or decisions made during the meta-analysis to detect any significant changes in the overall estimate. Additionally, potential publication bias for these continuous outcomes was visually assessed by generating and examining funnel plots using RevMan 5.4, with any asymmetry in the plot investigated as a potential indicator of bias. Prediction intervals indicated the expected range of true effects in future patient populations^[27]^. Simultaneously, for various complication rates, the proportion data from the included studies were subjected to a logit transformation. These transformed proportions were then combined using an inverse variance-weighted fixed-effects model. Ultimately, the comparative effectiveness for both continuous outcomes and complication rates was evaluated through subgroup analyses conducted in R software (version 4.4.3) using the “meta” package. Detailed results from individual studies and the pooled effects for each subgroup are presented in the forest plots. A *P* value of less than 0.05 was considered statistically significant^[28]^.

## Results

### Study and patient characteristics

Studies published until October 2024 were reviewed, encompassing 1810 studies. After excluding 344 duplicates, 1466 studies remained. Title and abstract screenings excluded 1381 articles based on the preset criteria. The full-text assessments of the remaining 85 studies identified 24 eligible studies, as shown in the PRISMA flowchart (Fig. 1). Among these, 13 were cohort studies, 10 were case series, and one was an RCT.

This analysis included 956 shoulders. The MA group encompassed 5 studies with 206 shoulders, averaging 64.58 ± 8.80 years in age with a mean follow-up of 19.68 ± 8.56 months (Range:12-70 months). The LDTT group included 19 studies with 750 shoulders, averaging 60.84 ± 7.55 years in age with a mean follow-up of 31.15 ± 13.07 months (Range:12-126 months). Study characteristics and demographic data are summarized in Tables 1 and 2, respectively.

**Table 1:** Study details

First author	Year	Level of evidence; study type	Surgery approach	Type of graft	No. of shoulders	Age, y			Follow-up, mo			Sex	
						Mean	SD	Range	Mean	SD	Range	Male	Female
**MA**													
Gupta *et al*^[27]^	2024	4; Retrospective case series	All-arthroscopic	No	43	56.2	6.2	42–70	18.8	11.3	12–55	29	9
Oh *et al*^[28]^	2024	3; Cohort study	Arthroscopically assisted	No	29	62.6	6.9		12	0		12	17
Yokoya *et al* (study group)^[29]^	2020	3; Cohort study	Arthroscopically assisted	PGA	47	68.3	8.1		24.2	1		27	20
Yokoya *et al* (control group)^[29]^	2020	3; Cohort study	Arthroscopically assisted	No	27	69.4	7.1		24.3	0.7		12	15
Yokoya *et al*^[20]^	2019	3; Retrospective cohort study	Arthroscopically assisted	No	26	68.6	8.8		28.3	7.3		13	13
Morihara *et al*^[22]^	2018	NR	Arthroscopically assisted	No	34	64.8	6.9	46–73	32.5		24–70	21	12
**LDTT**													
Kany *et al* (all-arthroscopic)^[30]^	2024	3; Retrospective cohort comparison treatment study	All-arthroscopic	Autograft	31	61	9.53	42.7–80.5	30.6	8.2	24–51	16	15
Kany *et al* (arthroscopically assisted)^[30]^	2024	3; Retrospective cohort comparison treatment study	Arthroscopically assisted	Autograft	31	63.1	10.66	43.1–81.4	33.3	8.8	24–62	18	13
Kany *et al* (arthroscopically assisted)^[31]^	2023	3; Cohort study	Arthroscopically assisted	Autograft	31	63.8	8.29	53.0–79.8	41	13.9	24–66	20	11
Kany *et al* (all-arthroscopic)^[31]^	2023	3; Cohort study	All-arthroscopic	Autograft	31	66	7.7	50.2–80.7	36	8.3	24–49	17	14
Reinares *et al*^[32]^	2022	4; Prospective cohort study	Arthroscopically assisted	Autograft	15	53.4			37	16		8	7
Baek *et al*^[33]^	2022	3; Retrospective cohort comparison treatment study	Arthroscopically assisted	Autograft	48	60.9	4.5		24			32	16
Ozturk *et al*^[34]^	2021	2; Randomized controlled trial treatment study	Arthroscopically assisted	Autograft	21	63.7	6.7		32.8	5.8		8	13
Kany *et al* (patients ≤55 years of age)^[35]^	2021	3; Retrospective cohort comparison treatment study	Arthroscopically assisted	Autograft	31	52		43–55	33	13		35	27
Kany *et al* (patients >55 years of age)^[35]^	2021	3; Retrospective cohort comparison treatment study	Arthroscopically assisted	Autograft	31	77		75–83	31	9.6			
Waltenspül *et al*^[36]^	2021	4; Case series treatment study	Arthroscopically assisted	Autograft	31	55.5		38–73	42		24–60	23	8
Baverel *et al*^[37]^	2021	3; Retrospective cohort comparison treatment study	Arthroscopically assisted	Autograft	21	65.4	9.5	52–84	22.1	4.1	12–30	11	10
Woodmass *et al*^[38]^	2020	4; A Multicenter retrospective analysis	Arthroscopically assisted	Autograft	16	57	6		22	10		13	3
Elhassan *et al*^[39]^	2020	4; Case series treatment study	Arthroscopically assisted	Autograft	56	53		33–79	13		7–51	17	39
Valenti *et al* (isolated transfer)^[40]^	2019	2; Treatment study (prospective cohort design)	Arthroscopically assisted	Autograft	17	58			22		18–34	9	8
Valenti *et al* (transfer combined with a partial cuff repair)^[40]^	2019	2; Treatment study (prospective cohort design)	Arthroscopically assisted	Autograft	14	53			22		18–34	9	5
Pogorzelski *et al*^[41]^	2018	4; Retrospective case series	Arthroscopically assisted	Allograft	16	49		26–57	66		25.2–126	10	6
Kany *et al*^[42]^	2018	4; Case series treatment study	Arthroscopically assisted	Autograft	60	64.2		46–83	35.2		24–50	26	34
Cavalier *et al*^[43]^	2018	3; Non-randomized prospective study	Arthroscopically assisted	Autograft	25	65			12			12	13
Yamakado *et al*^[44]^	2017	4; Therapeutic case series	Arthroscopically assisted	Autograft	30	67.4	6.2	54–78	34		24–72	22	8
Petriccioli *et al*^[45]^	2016	4; Case series treatment study	Arthroscopically assisted	Autograft	33	57.9		31–69	35.7		12–60	22	11
Castricini *et al*^[46]^	2016	4; Case series treatment study	Arthroscopically assisted	Autograft	86	59.8	5.9	38–69	36.4	9	24–60	48	38
Paribelli *et al*^[47]^	2015	Prospective case–control study	Arthroscopically assisted	Autograft	20	62.5		45–77	33.6	36	12–60	13	7
Grimberg *et al*^[48]^	2015	4; Therapeutic case series	Arthroscopically assisted	Autograft	55	62		31–75	29.4		24–42	25	30

LDTT, latissimus dorsi tendon transfer; MA, muscle.

**Table 2:** Study characteristics at pre-operation

Outcome	MA	LDT	*P* value
	Mean ± SD	Mean ± SD	
Age, y	64.58 ± 8.80	60.84 ± 7.55	<0.001
Follow-up, mo	**19.68 ± 8.56**	**31.15 ± 13.07**	**<0.001**
Sex (M/F)	**114/86**	**414/336**	**0.649**
No. of shoulder	**206**	**750**	

LDTT, latissimus dorsi tendon transfer; MA, muscle advancement; F, female; M, male; SD, standard deviation; mo, months; y, years old.

Bold values indicate statistical significance.

Sensitivity analyses showed no significant changes in the pooled effect estimates, 95% confidence intervals (CIs), or statistical significance for the primary outcome measures. This indicates that the main findings of this study are robust to methodological variations. Additionally, funnel plots for most primary outcome measures displayed a generally symmetrical distribution around the vertical line, representing the pooled effect estimate. This suggests a low likelihood of significant publication bias for the primary outcomes assessed in this study (Supplemental Digital Content, available at: http://links.lww.com/JS9/E632).

### Risk of bias assessment

The overall methodological quality of the included studies is acceptable. The randomized controlled trial demonstrated a low risk of bias in most domains, with only some concerns regarding the blinding of outcome assessment. Non-randomized studies were predominantly rated as having a moderate overall risk of bias, which was mainly attributable to residual confounding and limitations in the participant selection. Despite these issues, the risk of bias related to outcome measurement and selective reporting was consistently low, indicating a reasonable level of reliability of the reported results (Fig. 2).

**Figure 2.**
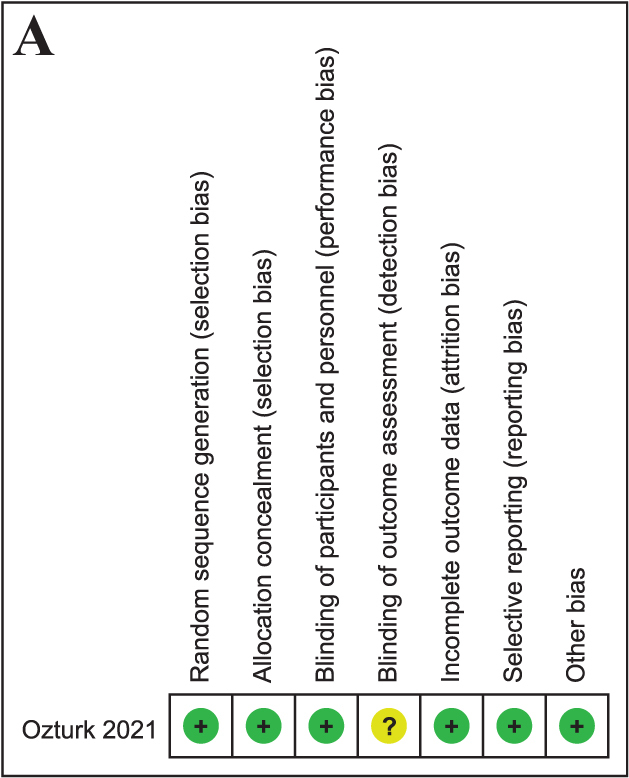

**Figure 2.**
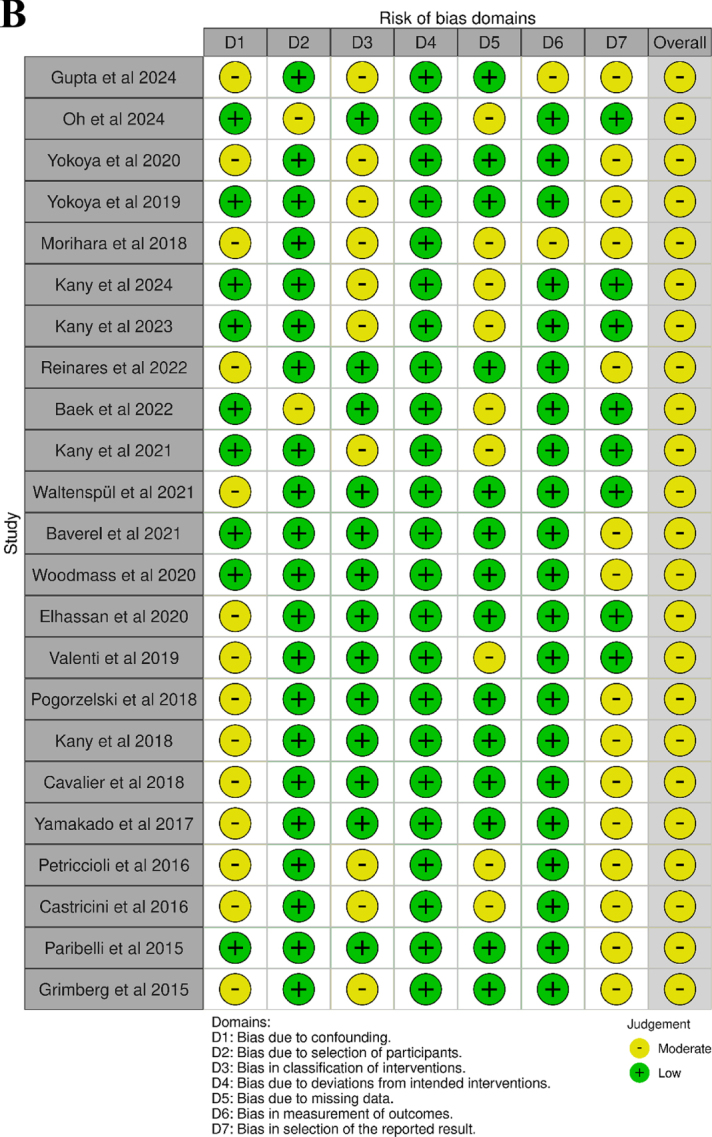
Risk of bias assessment results using (A) RoB 2 tool for RCT studies and (B) the ROBINS-I tool for non-RCT studies.

### Surgical techniques

In the MA group, one study utilized an all-arthroscopic approach, while four employed arthroscopy-assisted methods^[29]^. Owing to the nature of MA surgery, which entails freeing and advancing muscles to reduce tension and facilitate repair, a graft is typically unnecessary^[20,22,30,31]^. However, one cohort study used a polyglycolic acid (PGA) sheet to support rotator cuff repair and promote tissue regeneration, highlighting a unique approach intended to enhance surgical outcomes^[31]^.

All surgical techniques in the LDTT group were either arthroscopically assisted or fully arthroscopic^[32–50]^. Two comparative studies specifically employed total arthroscopic LDTT based on Kany’s method^[32,33]^. Most studies used an autograft Achilles tendon graft^[32–42,44–50]^, except for one that used a different allograft (Table 1)^[43]^.

### Functional outcomes

#### Constant-Murley score

**Figure 3.**
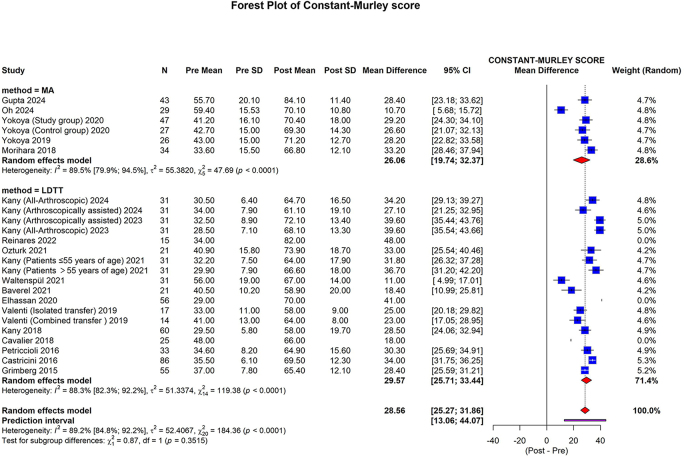
Forest plot of the subgroup analysis comparing MA and LDTT in CMS.MA, muscle advancement; LDTT, latissimus dorsi tendon transfer; CI, confidence interval; SD, standard deviation; CMS, constant-murley score.

CMS was reported in 5 studies (206 shoulders) in the MA group^[20,22,29–31]^ and 14 studies (620 shoulders) in the LDTT group^[32–34,36–39,41,42,44,45,47,48,50]^. The pooled mean difference (in the MA group) was 26.06 (95% CI: 19.74 to 32.37; I2 = 89.5%; *P* < 0.0001). The pooled mean difference (in the LDTT group) was 29.57 (95% CI: 25.71 to 33.44; I2 = 88.3%; *P* < 0.0001). The overall pooled mean difference was 28.56 (95% CI: 25.27 to 31.86; 95% prediction interval: 13.06 to 44.07; I2 = 89.2%, *P* < 0.0001). No statistically significant differences were observed between the subgroups (*P* = 0.3515) (Fig. 3).

#### UCLA score

**Figure 4.**
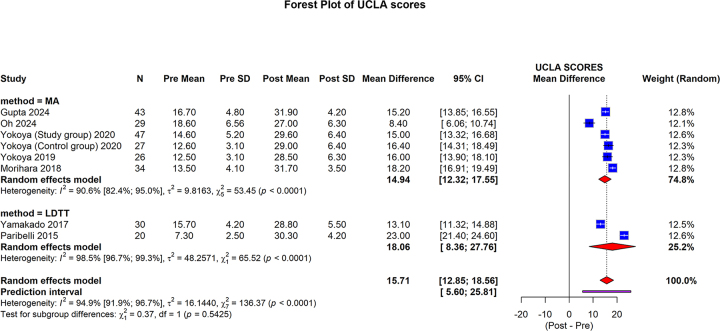
Forest plot of subgroup analysis comparing MA and LDTT in the UCLA score.

MA, muscle advancement; LDTT, latissimus dorsi tendon transfer; CI, confidence interval; SD, standard deviation

The UCLA Shoulder Score was reported in five studies (206 shoulders) in the MA group^[20,22,29–31]^ and two studies (50 shoulders) in the LDTT group^[46,49]^. The MA group, pooled mean difference was 14.94 (95% CI: 12.32 to 17.55; I2 = 90.6%, *P* < 0.0001). In the LDTT group, the pooled mean difference was 18.06 (95% CI: 8.36 to 27.76; I2 = 98.5%, *P* < 0.0001). The overall pooled mean difference was 15.71 (95% CI: 12.85 to 18.56; 95% prediction interval: 5.60 to 25.81; I2 = 94.9%; *P* < 0.0001). No statistically significant differences were observed between the subgroups (*P* = 0.5425) (Fig. 4).

#### ASES score

**Figure 5.**
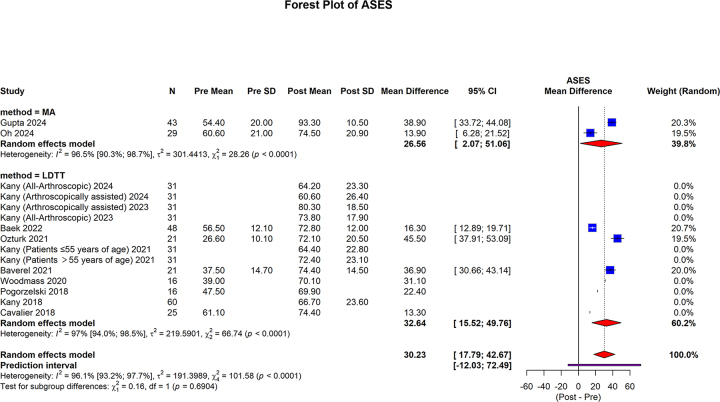
Forest plot of subgroup analysis comparing MA and LDTT in ASES score.

MA, muscle advancement; LDTT, latissimus dorsi tendon transfer; CI, confidence interval; SD, standard deviation; ASES, American Shoulder and Elbow Surgeons

ASES scores were reported in two studies (72 shoulders) in the MA group^[29,30]^ and ten studies (393 shoulders) in the LDTT group^[32,33,35–37,39,40,43–45]^. In the MA group, the pooled mean difference was 26.56 (95% CI: 2.07 to 51.06; I2 = 96.5%, *P* < 0.0001). In the LDTT group, the pooled mean difference was 32.64 (95% CI: 15.52 to 49.76; I2 = 97.0%, *P* < 0.0001). The overall pooled mean difference was 30.23 (95% CI: 17.79 to 42.67; 95% prediction interval: −12.03 to 72.49; I2 = 96.1%, *P* < 0.0001). No statistically significant difference was observed between the subgroups (*P* = 0.6904) (Fig. 5).

#### VAS pain score

**Figure 6.**
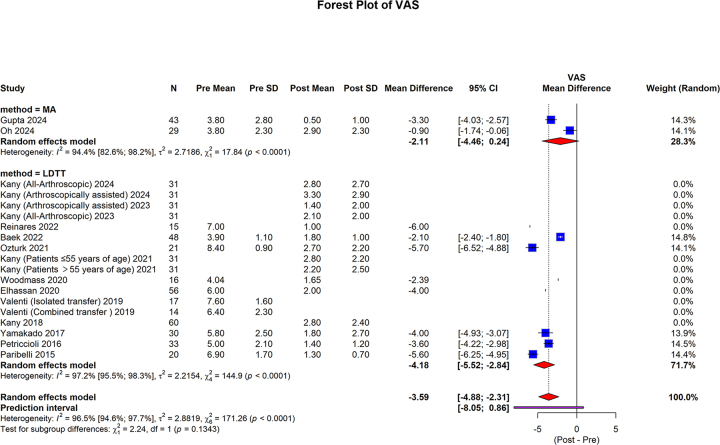
Forest plot of subgroup analysis comparing the MA and LDTT VAS scores.

MA, muscle advancement; LDTT, latissimus dorsi tendon transfer; CI, confidence interval; SD, standard deviation VAS, visual analog scale

The VAS pain scores, measured in 72 cases in the MA group^[29,30]^ and 485 cases in the LDTT group^[32–37,40,41,44,46,47,49]^, showed that in the MA group, pooled mean difference was −2.11 (95% CI: −4.46 to 0.24; I2 = 94.4%, *P* < 0.0001). The LDTT group pooled mean difference was −4.18 (95% CI: −5.52 to −2.84; I2 = 97.2%, *P* < 0.0001). The overall pooled mean difference was −3.59 (95% CI: −4.88 to −2.31; 95% prediction interval: −8.05 to 0.86; I2 = 96.5%, *P* < 0.0001). No statistically significant differences were observed between the subgroups (*P* = 0.1343) (Fig. 6).

#### Radiological evaluation

**Figure 7.**
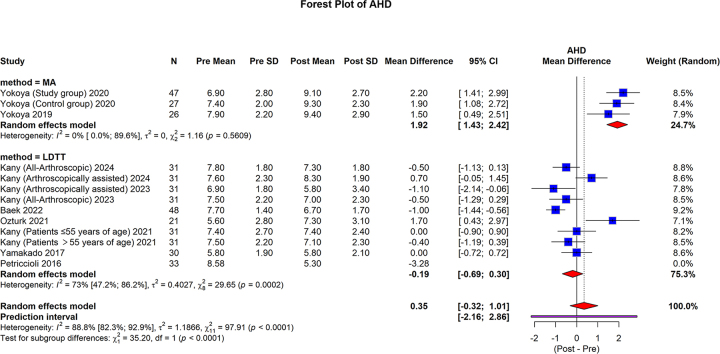
Forest plot of subgroup analysis comparing MA and LDTT in AHD.

MA, muscle advancement; LDTT, latissimus dorsi tendon transfer; CI, confidence interval; SD, standard deviation; AHD, acromiohumeral distance

The AHD, measured in 100 cases in the MA group^[20,22,31]^ and 318 cases in the LDTT group^[32,33,35–37,46,47]^, showed that MA group pooled mean difference was 1.92 (95% CI: 1.43 to 2.42; I2 = 0%, *P* = 0.5609). The LDTT group pooled mean difference was −0.19 (95% CI: −0.69 to 0.30; I2 = 73%, *P* = 0.0002). The overall pooled mean difference was 0.35 (95% CI: −0.32 to 1.01; 95% prediction interval: −2.16 to 2.86, I2 = 88.8%, *P* < 0.0001). The MA group showed a statistically significantly higher pooled mean difference than the LDTT group (*P* < 0.0001) (Fig. 7).

#### ROM

Abduction was measured in two studies (77 cases, MA group)^[22,29]^ and ten studies (510 cases, LDTT group)^[32–34,36–38,41,44,48,50]^. FF was measured in four studies (177 cases, MA group)^[20,22,29,31]^ and 18 studies (734 cases, LDTT group)^[32–42,44–50]^. ER was measured in five studies (206 cases, MA group)^[20,22,29,31]^ and 17 studies (678 cases, LDTT group)^[32–42,44–50]^.

**Figure 8.**
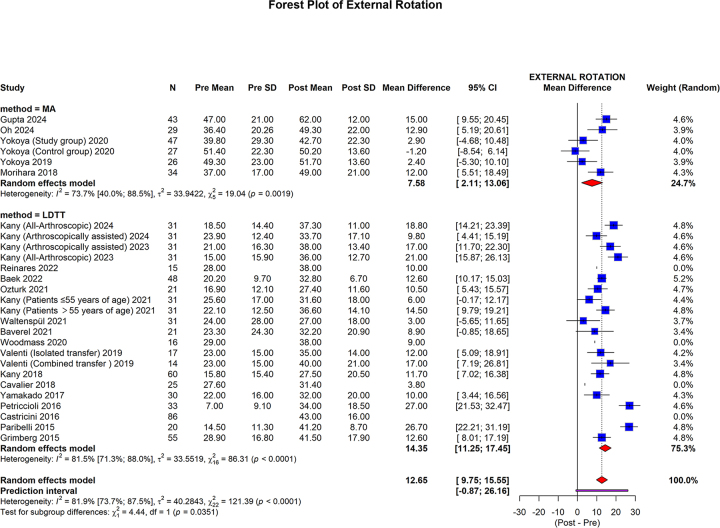
Forest plot of subgroup analysis comparing MA and LDTT in the ER.

MA, muscle advancement; LDTT, latissimus dorsi tendon transfer; CI, confidence interval; SD, standard deviation; ER, external rotation

ER showed that MA group pooled mean difference was 7.58 (95% CI: 2.11 to 13.06; I2 = 73.7%, *P* = 0.0019). In the LDTT group, the pooled mean difference was 14.35 (95% CI: 11.25 to 17.45; I2 = 81.5%, *P* < 0.0001). The overall pooled mean difference was 12.65 (95% CI: 9.75 to 15.55; 95% prediction interval: −0.87 to 26.16; I2 = 81.9%, *P* < 0.0001). The LDTT group showed a significantly higherpooled mean difference than the MA group (*P* = 0.0351) (Fig. 8).

**Figure 9.**
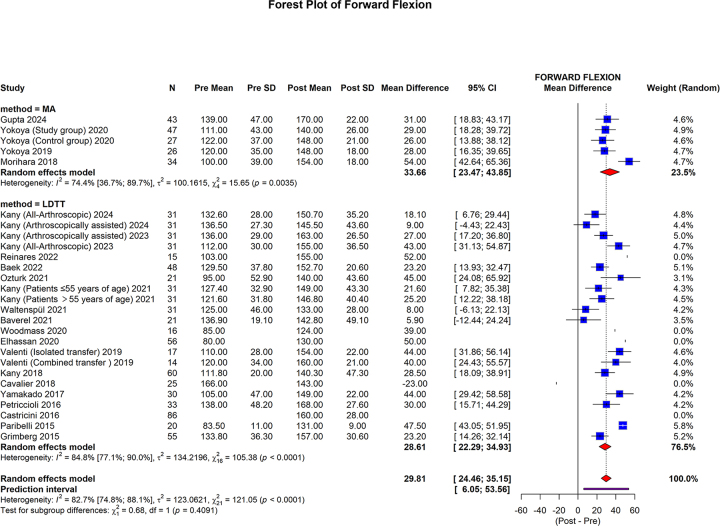
Forest plot of the subgroup analysis comparing MA and LDTT in FF.

MA, muscle advancement; LDTT, latissimus dorsi tendon transfer; CI, confidence interval; SD, standard deviation; FF, forward flexion

The FF showed that in the MA group, the pooled mean difference was 33.66 (95% CI: 23.47 to 43.85; I2 = 74.4%, *P* = 0.0015). In the LDTT group, the pooled mean difference was 28.61 (95% CI: 22.26 to 34.93; I2 = 84.8%, *P* < 0.0001). The overall pooled mean difference was 29.81 (95% CI: 24.46 to 35.16; 95% prediction interval: 6.05 to 53.56; I2 = 82.7%, *P* < 0.0001). No statistically significant differences were observed between the subgroups (*P* = 0.4081) (Fig. 9).

**Figure 10.**
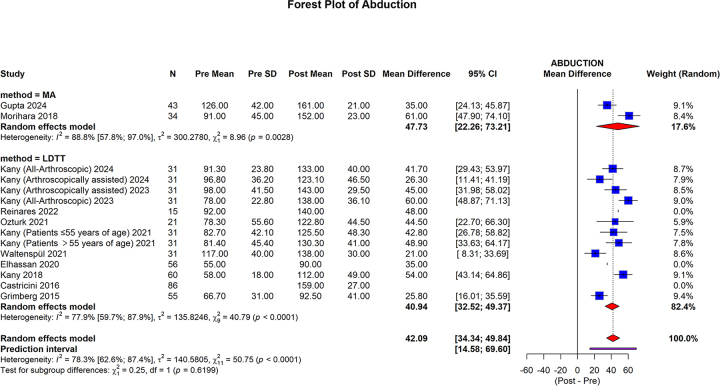
Forest plot of the subgroup analysis comparing MA and LDTT in abduction.

MA, muscle advancement; LDTT, latissimus dorsi tendon transfer; CI, confidence interval; SD, standard deviation

The abduction showed that MA group pooled mean difference was 47.73 (95% CI: 22.26 to 73.21; I2 = 88.8%, *P* = 0.0028). In the LDTT group, pooled mean difference was 40.94 (95% CI: 32.52 to 49.37; I2 = 77.9%, *P* < 0.0001). The overall pooled mean difference was 42.09 (95% CI: 34.34 to 49.84; 95% prediction interval: 14.58 to 69.60; I2 = 78.3%, *P* < 0.0001). No statistically significant differences were observed between the subgroups (*P* = 0.6199) (Fig. 10).

## Complications

**Figure 11.**
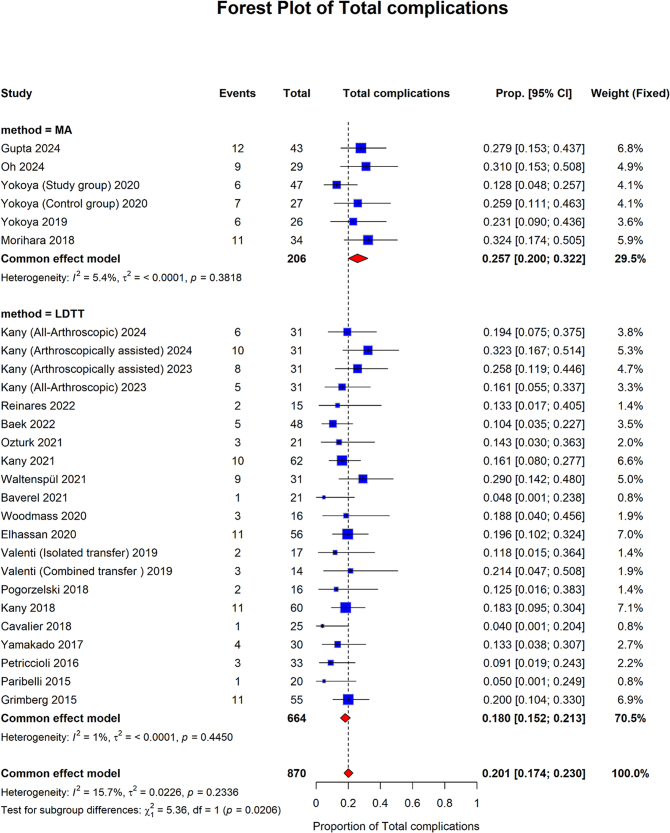
Forest plot of subgroup analysis comparing MA and LDTT for complications.

MA, muscle advancement; LDTT, latissimus dorsi tendon transfer; CI, confidence interval

Total complications showed that the pooled proportion of the MA group was 0.257 (95% CI: 0.200–0.322; I2 = 5.4%, *P* = 0.3818). The LDTT pooled proportion was 0.180 (95% CI: 0.152 to 0.213; I2 = 1%, *P* = 0.4450). The overall pooled proportion was 0.201 (95% CI: 0.174 to 0.230; I2 = 15.7%, *P* = 0.2336). The MA group showed a statistically significantly higher pooled proportion than the LDTT group (*P* = 0.0206) (Fig. 11).

**Figure 12.**
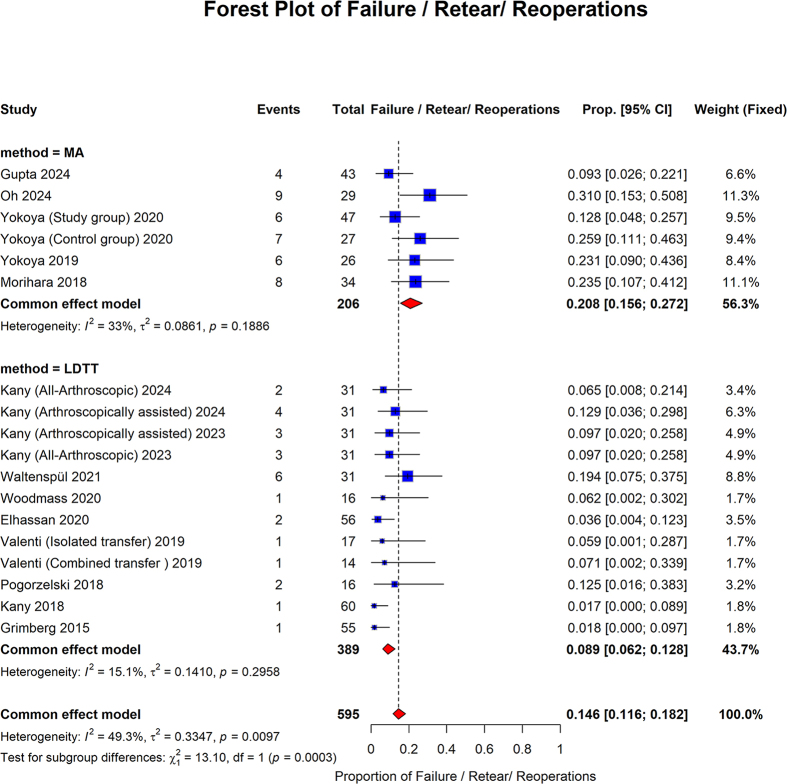
Forest plot of subgroup analysis comparing MA and LDTT in retear and reoperation.

MA, muscle advancement; LDTT, latissimus dorsi tendon transfer; CI, confidence interval

Failure/retear/reoperations showed that the pooled proportion of the MA group was 0.208 (95% CI, 0.156–0.272; I2 = 33%, *P* = 0.1886). The LDTT group pooled proportion was 0.089 (95% CI: 0.062 to 0.128; I2 = 15.1%, *P* = 0.2958). The overall pooled proportion was 0.146 (95% CI: 0.116 to 0.182; I2 = 49.3%, *P* = 0.0097). The MA group showed a significantly higher pooled proportion of Failure/Retear/Reoperations compared to the LDTT group (*P* = 0.0003) (Fig. 12).

**Figure 13.**
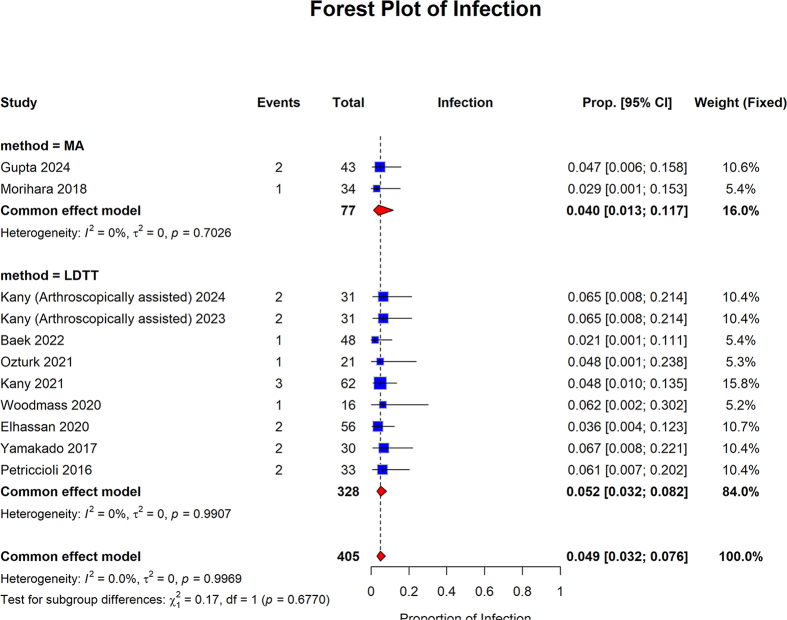
Forest plot of the subgroup analysis comparing MA and LDTT in infection.

MA, muscle advancement; LDTT, latissimus dorsi tendon transfer; CI, confidence interval

The pooled proportion of the MA group was 0.040 (95% CI: 0.013–0.117; I2 = 0%, *P* = 0.7026). The pooled proportion of patients in the LDTT group was 0.052 (95% CI: 0.032–0.082; I2 = 0%, *P* = 0.9907). The overall pooled proportion was 0.049 (95% CI: 0.032 to 0.076; I2 = 0.0%, *P* = 0.9969). No statistically significant differences were observed between the subgroups (*P* = 0.6770) (Fig. 13).

**Figure 14.**
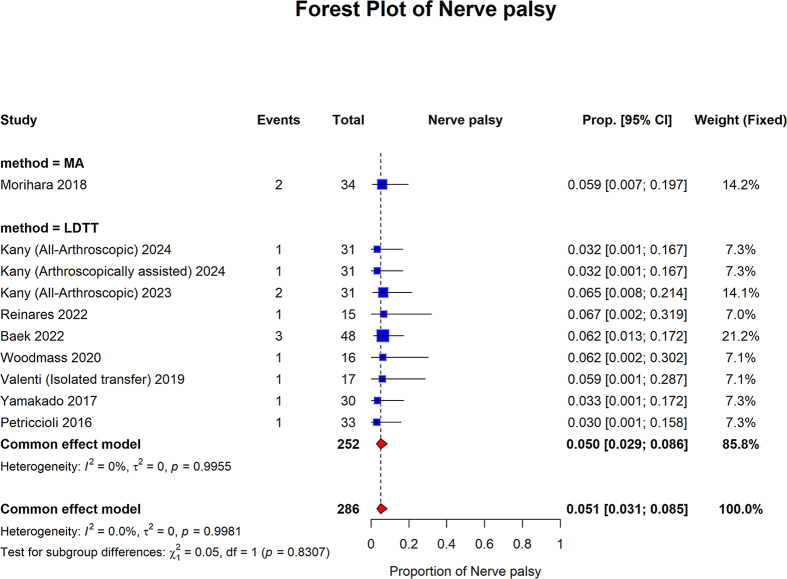
Forest plot of subgroup analysis comparing MA and LDTT in nerve palsy.

MA, muscle advancement; LDTT, latissimus dorsi tendon transfer; CI, confidence interval

The proportion of patients with nerve palsy in the MA group was 0.059 (95% CI: 0.007–0.197). The pooled proportion of the LDTT group was 0.050 (95% confidence interval [CI]: 0.029–0.086; I2 = 0%, *P* = 0.9955). The overall pooled proportion was 0.051 (95% CI: 0.031 to 0.085; I2 = 0.0%, *P* = 0.9981). No statistically significant differences were observed between the subgroups (*P* = 0.8307) (Fig. 14).

**Figure 15.**
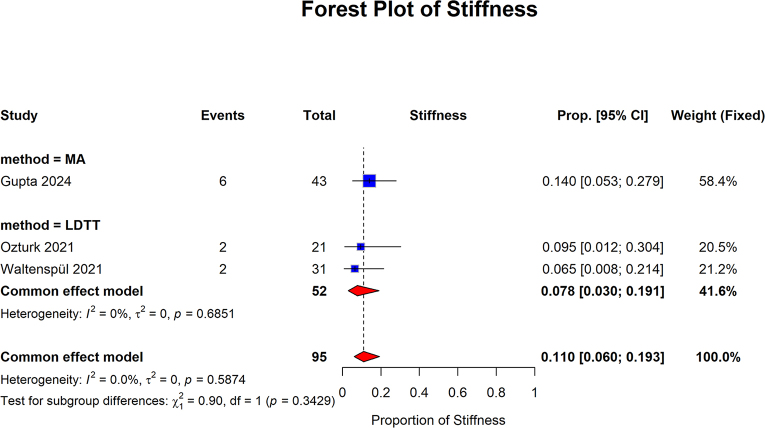
Stiffness Forest plot of subgroup analysis comparing MA and LDTT in stiffness.

MA, muscle advancement; LDTT, latissimus dorsi tendon transfer; CI, confidence interval

The stiffness showed that the proportion of patients in the MA group was 0.140 (95% CI: 0.053–0.279). The pooled proportion of the LDTT group was 0.078 (95% confidence interval [CI]: 0.030–0.191; I2 = 0%, *P* = 0.6851). The overall pooled proportion was 0.110 (95% CI: 0.060 to 0.193; I2 = 0.0%, *P* = 0.5874). No statistically significant differences were observed between the subgroups (*P* = 0.3429) (Fig. 15).

The complications observed in the LDTT group comprised complete graft rupture (12 cases), seroma (31 cases), lymphedema (1 case), osteoarthritis (1 case), wound dehiscence (2 cases), fracture of the greater tuberosity (4 cases), and asymptomatic granuloma (1 case).

## Discussion

This systematic review analyzed 24 studies involving 956 cases of rotator cuff injuries treated with either LDTT or MA. The LDTT group consisted of 750 cases across 19 studies^[32–50]^, while the MA group included 206 cases from 5 studies^[20,22,29–31]^. Comparative analysis revealed differences in clinical outcomes, functional recovery, and complications between the two surgical techniques. This study also provides a critical addition to the existing literature by addressing a key gap in which comparative data were previously limited by presenting the first comprehensive meta-analysis to directly compare these two techniques. Our findings establish that the optimal choice is not about one technique’s overall superiority but rather a nuanced decision based on a trade-off between biomechanical advantages and distinct risk profiles.

The core differences between the MA and LDTT stem from their respective anatomical repair mechanisms and biomechanical objectives. MA involves advancing the supraspinatus and infraspinatus muscles in situ to directly repair the torn area, restoring the original anatomical structure and force couple balance of the rotator cuff while maintaining tension-free repair, which is crucial for reducing the risk of retear and promoting healing^[51–53]^. In the short term, shoulder joint stability is achieved through static tension restoration. In contrast, LDTT transfers the latissimus dorsi tendon to the greater tuberosity, converting it from an internal to an external rotator, dynamically reconstructing the posterior-superior force couple to compensate for the rotator cuff defect^[54]^. Although this compensatory mechanism cannot fully restore the original anatomical structure, it dynamically covers the humeral head defect, leading to long-term improvement in external rotation. Therefore, MA may rely on muscle quality for anatomical repair, whereas LDTT may be more suitable for end-stage injuries through biomechanical compensation.

The complication profiles of MA and LDTT differ because of their distinct surgical principles. MA showed higher overall (25.7% vs. 18.0%) and retear rates (20.8 % vs. 8.9%), reflecting its reliance on native tissue repair in older patients (64.58 vs 60.84 years). Conversely, the LDTT tendon transfer approach avoids these issues but introduces graft-specific complications (ruptures, seromas, and tuberosity fractures). This dichotomy highlights MA’s tissue-dependent limitations of MA versus graft-related risks of LDTT, emphasizing the need for individualized surgical selection based on patient factors and repair objectives. Clinicians must weigh the key functional trade-off between the two techniques. While Muscle Advancement (MA) offers superior static joint stability with greater improvement in AHD, Latissimus Dorsi Tendon Transfer (LDTT) effectively restores dynamic external rotation. This decision is further complicated by MA’s higher risk of retear and reoperation of MA, which must be balanced against the unique graft-related complications of LDTT.

MA may be more suitable for patients with preserved muscle quality, higher baseline function, and those seeking rapid recovery, particularly young and active individuals. In contrast, LDTT could be considered more appropriate for end-stage injuries, patients with poor baseline function, and those requiring long-term biomechanical compensation, especially those with chronic pain and severe external rotation deficits. Future research might explore modified techniques to integrate the advantages of anatomical and biomechanical repair.

In this meta-analysis, significant heterogeneity was evident across continuous clinical outcomes, necessitating the use of random effects models for data synthesis. This substantial variability is primarily due to the nature of the included evidence, which predominantly consists of observational studies and lacks high-quality randomized controlled trials (RCTs). The inherent limitations of non-randomized designs, such as selection bias and confounding, are key contributors to the inconsistencies observed between study results. Several methodological steps were undertaken to rigorously address and investigate this heterogeneity. We conducted a systematic quality assessment of all included studies using established tools, such as ROBINS-I and Cochrane’s risk-of-bias tool. Sensitivity analyses, specifically leave-one-out analyses, were performed to evaluate the robustness of the pooled estimates despite the variability. Additionally, funnel plots were generated and examined to assess potential publication bias, which could contribute to heterogeneity. These efforts provided a clearer context for interpreting our findings. Beyond the study design quality, clinical and methodological diversity, including variations in patient characteristics, follow-up durations, and specific surgical techniques, likely further contributed to this heterogeneity. Consequently, a cautious interpretation of the pooled effect estimates is warranted, recognizing that the average effect may not apply uniformly. Future research, particularly well-designed and meticulously reported RCTs, is crucial to generate more consistent and reliable clinical evidence.

## Conclusions

LDTT and MA effectively restore shoulder function in IRCTs; however, their mechanisms differ. The LDTT excels in dynamic biomechanical compensation for external rotation, whereas MA achieves superior static joint stability. LDTT’s lower retear rates and higher complication risks associated with MA highlight the need for patient-specific surgical selection.

## Data Availability

The raw data could be available for scientific purpose by sending requests to the corresponding author.

## Research registration unique identifying number (UIN)

The study protocol was registered in PROSPERO (CRD42024617366).
